# TRP channels and monoterpenes: Past and current leads on analgesic properties

**DOI:** 10.3389/fnmol.2022.945450

**Published:** 2022-07-29

**Authors:** Hugues Petitjean, Eléa Héberlé, Louis Hilfiger, Olga Łapieś, Guillaume Rodrigue, Alexandre Charlet

**Affiliations:** ^1^Benephyt, Strasbourg, France; ^2^Centre National de la Recherche Scientifique, University of Strasbourg, Institute of Cellular and Integrative Neuroscience, INCI UPR3212, Strasbourg, France

**Keywords:** monoterpenes, pain, inflammation, TRP, pulegone, menthol, hyperalgesia

## Abstract

The activation of the transient receptor potential (TRP) channels expressed by sensory neurons is essential to the transduction of thermal and mechanical sensory information. In the setting of chronic inflammatory conditions, the activation of the melastatin family member 8 (TRPM8), the TRP vanilloid 1 (TRPV1), and the TRP ankyrin 1 (TRPA1) is correlated with pain hypersensitivity reactions. Monoterpenes, among which pulegone and menthol, a major class of phytocompounds present in essential oils of medicinal plants, are known modulators of those TRP channels activity. In the present review, we correlate the monoterpene content of plants with their historical therapeutic properties. We then describe how monoterpenes exert their anti-inflammatory and antihyperalgesia effects through modulation of TRP channels activity. Finally, we discuss the importance and the potential of characterizing new plant extracts and reassessing studied plant extracts for the development of ethnopharmacology-based innovative treatments for chronic pain. This review suggests that monoterpene solutions, based on composition from traditional healing herbs, offer an interesting avenue for the development of new phytotherapeutic treatments to alleviate chronic inflammatory pain conditions.

## Introduction

Chronic inflammatory pain conditions such as osteoarthritis are debilitating diseases that impair the normal life of 14–36% of the population of the USA (Neogi, 2013; Treede et al., 2019). They are characterized by the presence of spontaneous pain, not evenly linked to an injury, and by hypersensitivity to painful stimuli (Raja et al., 2020). Despite the existence of several medications to alleviate chronic pain conditions, such as opioid treatment, which come with their share of unwanted side effects (Opioid Overdose Crisis | National Institute on Drug Abuse, 2022), most debilitating chronic inflammatory states remain uncured (Gilbert, 2021). Phytotherapy, or traditional herbalism, referring to the use of plant-derived preparation in the treatment and prevention of disease, might be a promising lead, with lower rates of adverse effects and efficiencies that might reach those of currently used conventional drugs (Dragos et al., 2017). Across the world, it remains the first intentional solution to diseases, or a preponderant complement to “conventional” medicine. Phytotherapy has been increasingly supported by the WHO (World Health Organization, 2013), and has been used to treat chronic joint inflammatory disorders such as osteoarthritis (Cameron and Chrubasik, 2014), rheumatoid arthritis (Dragos et al., 2017), and to alleviate chronic low back pain (Oltean et al., 2014).

Regarding their elaborate secondary metabolism, plants are indeed interesting reservoirs of phytocompounds with pharmacological activities (Yang et al., 2018). Among secondary metabolites, monoterpenes, such as menthol, are small volatile and fragrant molecules synthesized through the terpenoid biosynthesis pathway (Bergman and Phillips, 2021). Cyclohexanoids monoterpenes, also known as “p-menthanes,” are highly valuable molecules in industry and of important economical value, accounting for the majority of the compounds in essential oils (Tetali, 2018; Bergman et al., 2019). Widespread across the plant kingdom, menthol is, among this compound family, the most studied. It represents a reference molecule (Croteau et al., 2005), especially in the case of the modulation of pain conditions (Galeotti et al., 2002). Menthol is the naturally cyclic monoterpene alcohol found in the peppermint plant *Mentha x piperita* L. that gives the *Mentha* species their distinctive peppermint odor and flavor, and this essential oil triggers cold sensation through the activation of the transient receptor potential (TRP) melastatin family member 8 (TRPM8) channel (Peier et al., 2002). Menthol not only modulates TRPM8 but also several receptors and channels such as other TRP channels [for review (Oz et al., 2017)]; notably the TRP vanilloid 1 (TRPV1) (Takaishi et al., 2014) and the TRP ankyrin 1 (TRPA1) (Karashima et al., 2007) that are both transducers of noxious stimulation and therefore contribute to pain hypersensitivities during inflammation (Julius, 2013). Despite the fact that terpenes are the largest class of secondary metabolites, with over 50,000 compounds with interesting biological activities (such as antibacterial and anti-inflammatory) (Zielińska-Błajet and Feder-Kubis, 2020), less than thirty monoterpenes have been identified to promote analgesia and antihyperalgesia effects (Sousa and Pergentino, 2011; Guimarães et al., 2013; Gouveia et al., 2018). In a recent comparative study, menthol and its precursor, pulegone, have both been demonstrated to bear anti-inflammatory and antihyperalgesic properties in an inflammatory pain rodent model (Hilfiger et al., 2021) suggesting that plant extracts containing pulegone could help alleviate pain conditions, in a synergistic fashion.

In this review, first, we will list the plants containing menthol and pulegone, and enumerate their therapeutic properties mentioned in ethnobotanical data, to define a new plant group as matrices of interest. Second, we will summarize the evidence demonstrating the pain-killer properties of menthol and other monoterpenes. This will be further extended by the description of the current state of knowledge on the modulation of TRP channels by menthol and pulegone leading to reduced nociception. Finally, we will discuss the importance and the potential of characterizing new and reassessing studied plant extracts for the development of ethnopharmacology-based innovative treatments for chronic pain.

## A historic standpoint: Lamiaceae, pulegone, and menthol

**Figure 1:**
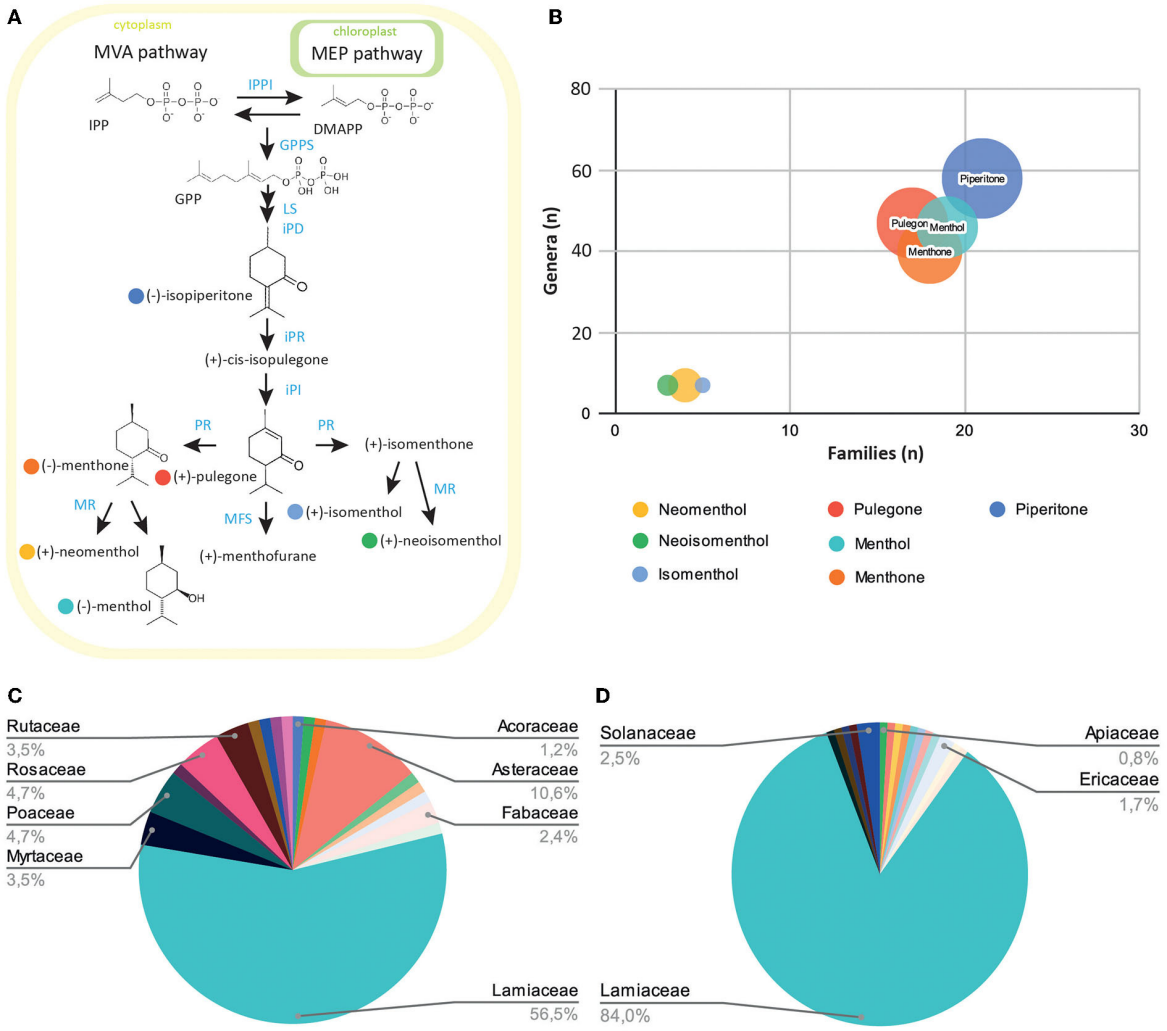
Exploration of the prevalence of menthol-like monoterpenes across the plant kingdom. **(A)** Schematic view of the monoterpene biosynthesis pathway leading to menthol, pulegone, and their derivatives. Plants produce the small precursor isopentenyl-pyrophosphate (IPP) through the mevalonate (MVA) pathway in the cytoplasm, or the methylerythritol (MEP) pathway in the plast. IPP can be isomerized into dimethylallyl-pyrophosphate (DMAPP) through the IPP isomerase activity (IPPI). Five-carbon units IPP and DMAPP can be converted into 10-unit geranyl-pyrophosphate (GPP) through the geranyl-pyrophosphate synthase (GPPS) activity. GPP is then converted into limonene, isopiperitone, and cis-isopulegone through limonene synthase (LS), trans-isopiperitenol dehydrogenase (iPD), and isopiperitone reductase (iPR) activity. Pulegone is then produced through cis-isopulegone isomerase (iPI) activity. Pulegone reductase (PR) converts pulegone into isomenthone or menthone, through different activities. These two compounds can then be converted into neomenthol and menthol, or isomenthol and neoisomenthol, respectively, through the menthone reductase activity (MR). The menthofurane synthase (MFS) can also convert pulegone into menthofurane, in an NADP-dependant reaction. **(B)** Bubble diagram representing the number of families (x-axis), genera (y-axis), and species (circle radius) containing monoterpenes precursors or derivatives. **(C)** Diagram of the distribution of menthol-containing plants within plant families. **(D)** Diagram of the distribution of pulegone-containing plants within plant families.

Menthol and pulegone are monoterpene derivatives synthesized through the conserved terpenoid backbone biosynthesis pathway (Figure 1A). They have been recently characterized and demonstrated to reduce the mechanical and thermal pain hypersensitivities in a rodent inflammatory pain model, thus reinforcing various previous results (de Sousa et al., 2007, 2011).

We first wanted to explore natural products databases to establish a list of plants currently known to contain menthol or pulegone. Because there are many natural products repositories (Sorokina and Steinbeck, 2020), we chose to investigate the LOTUS database, as it is one of the latest intents to harmonize, curate, and validate structure-organism pairs, in an open access fashion (Rutz et al., 2021). Dr. Duke's Phytochemical and Ethnobotanical Database (1992–2016), hosted on the USDA website (https://data.nal.usda.gov/dataset/dr-dukes-phytochemical-and-ethnobotanical-databases), was also consulted for its richness in ethnobotanical mentions for each plant. We started by extracting all plant names mentioned to contain pulegone and/or menthol, and also their precursor piperitone and their associated derivatives: neomenthol, isomenthol, and neoisomenthol (Figure 1B). To avoid any duplicates, their names were converted to their accurate scientific names and botanical families according to the Angiosperm Phylogeny Group IV (2016) and listed on “Plants of the World Online” (POWO, 2022 facilitated by the Royal Botanic Gardens; http://www.plantsoftheworldonline.org/—Retrieved 10 May 2022). Results show that piperitone appears in the highest number of species inventoried in the databases (over 158 species listed), throughout most of the plant families registered, whereas the derivatives of pulegone: (neomenthol, neoisomenthol, and isomenthol) present the lowest occurrence in the catalog (24, 13, and 12 species, respectively) (Figure 1B). The list of all plants recorded for the presence of menthol and pulegone forms a group of 85 and 110 species of plants, respectively (Supplementary Table 1). Among them, the Lamiaceae subgroup is best represented, with pulegone and menthol present in 84 and 56.5% of the Lamiaceae, respectively (Figures 1C,D).

Because of their aromatic properties, plants within this family have been extensively used across the world and for all kinds of purposes: food, aromatics, cosmetics, and healing. A trend to investigate plant properties based on their traditional use has emerged, embodied in the vast discipline of ethnopharmacology and showing promising results (Bruhn and Rivier, 2019). The re-examination of historical texts and traditional pharmacopeias will help to re-evaluate plant species' potential toward new indications that were discarded in the modern pharmacopeias (Heinrich et al., 2006).

During the Greek Antiquity (Leonti and Verpoorte, 2017), as referred to in Theophrastus' Historia Plantarum (Totelin, 2006), *Origanum majorana* and *Origanum sipylaeum* are mentioned as aromatics. *Mentha pulegium* is also mentioned with properties useful for pain treatment that were allegedly “known by all”: endemic from Crete and imported to Athens, it had a great reputation in ancient Greece (Totelin, 2006). In Mattioli's translation of Dioscorides' original texts from AD 50 to 70 (Mattioli, 1590), *Thymbrium* was cited as *Menta romana* for the Italians, and it was stated that leaves could relieve head pain when topically applied as a cataplasm. *Origanum* was described to relieve ear pain, when applied with milk. As for the “*Pulegium*,” probably referring to *M. pulegium* L., he stated that when applied with dried sour cherry, it relieved all inflammation and helped patients to support gout (Mattioli, 1590). A cross-observation between the following plants: *M. pulegium, Origanum*, and *M. romana*, reveals that they are all reported to contain menthol and pulegone; (Supplementary Table 1) eventually supporting a shared use based on their aromatic properties (Ntalli et al., 2010). Interestingly, 38 plants of the Lamiaceae family are present on the A list of the French Pharmacopeia, among which 22 are sold over the counter. Eight species are on the B list, due to their putative undesirable effects outnumbering the benefits they provide. Very few of them still have an indication for pain alleviation. As plant uses and medical paradigms fluctuate across history, sometimes resulting in loss of knowledge or transformations in the indications, it is important to re-assess plant compositions and their effects on a scientific basis.

Among those, *M. x piperita* L. is a textbook example, having been used for centuries in traditional medicine to reduce numerous ailments such as infections, insomnia, irritable bowel syndrome, and also pain (Farco and Grundmann, 2013). It has been demonstrated that the large quantity of menthol in this plant acts as the major active principle to alleviate joint pain (Topp et al., 2013). Another example is *Clinopodium nepeta* (L.) Kuntze, a plant of the Lamiaceae family, which has been mentioned since Greek Antiquity and in at least two of the most renowned herbals of the 16th and 17th centuries for the treatment of rheumatism (Adams et al., 2009): in Mattioli (1590), it is advised to boil and drink a *C. nepeta* (L.) Kuntze-based broth against gout and slime, while FUCHS (1543) recommends an external application of leaves against hip pain. In this case, the major active substance associated with beneficial effects is pulegone, which is found in large quantities in *C. nepeta* (L.) (BoŽović and Ragno, 2017). Recent evidence points out that pulegone, like menthol, acts as an antihyperalgesic phytocompound in an inflammatory pain animal model (Hilfiger et al., 2021).

Phytotherapy based on *M. x piperita* L. (*Mentha x officinalis* Hull) and *C. nepeta* (L.), Kuntze preparations rich in menthol or pulegone, as a major active compound, shows the potential for studying other members of the Lamiaceae family described throughout history of medicine for pain relief to reveal other analgesic monoterpenes. Chemotaxonomy suggests that other plants, which did not make it to the European traditional pharmacopeia, might also be interesting due to their composition. In our analysis, we found out, for instance, that *Clinopodium serpylifollium* subsp. fruticosum (L.) Bräuchler's [formally *Micromeria fruticosa* (L.) Druce] essential oil contains over 80% of pulegone, along with 36 other compounds. It might therefore be an interesting European plant to investigate (Kinmer et al., 1993). This idea is supported by the fact that essential oils from Lamiaceae do not exclusively contain pulegone or menthol but also other monoterpenes which, although present in low quantities, could act potentially in synergy on different molecular targets to alleviate pain (Maroon et al., 2010). However, as in the case of any molecule under investigation, it is crucial to assess toxicity effects (Wojtunik-Kulesza, 2022). Although pulegone's use as a food additive has been restricted by the FDA, there is no conclusive evidence of its carcinogenicity, and it is still authorized when extracted from natural sources (Food Drug Administration, 2018).

## Pulegone and menthol as potent analgesic and anti-inflammatory phytocompounds

Pulegone and menthol are both reported to have analgesic properties, along with 26 other monoterpenes and some of their derivatives (Guimarães et al., 2013; Wang et al., 2017; Dos Santos et al., 2021). To understand their putative pain relief properties, we reduced this group to monoterpenes associated with anti-inflammatory activity (de Cássia da Silveira e Sá et al., 2013) that is mediated at least through an anti-TNF-α activity (Chen et al., 2021; Hilfiger et al., 2021). We combined data reporting a modulation of the TRPV1, TRPA1, or TRPM8 activity to define a group of anti-inflammatory phytocompound modulators of TRP channels (Xu et al., 2005; de Sousa et al., 2010; Ortar et al., 2012; de Cássia da Silveira e Sá et al., 2013; Guimarães et al., 2013; Rufino et al., 2014; Takaishi et al., 2014; Mihara and Shibamoto, 2015; Ohtsubo et al., 2015; Dai, 2016; Kaimoto et al., 2016; Oz et al., 2017; Wang et al., 2017; Chamanara et al., 2019; de Christo Scherer et al., 2019; Quintans et al., 2019; Soleimani et al., 2019; Ghosh et al., 2020; Heblinski et al., 2020; Hilfiger et al., 2020; Islam et al., 2020; Zhu et al., 2020; Chen et al., 2021; Dos Santos et al., 2021; Kashiwadani et al., 2021) (Table 1). Both menthol and pulegone, among other monoterpenes, match these properties (Table 1). Interestingly, *M. pulegium* L. and *Origanum dictamnus* L. have been used in traditional pain-relief preparations and their essential oil analysis revealed that their essential oils contain 5 and 6 of those monoterpenes, respectively (Ntalli et al., 2010) (see Table 1).

**Table 1:** Anti-inflammatory monoterpenes modulating TRP channel activity compared to the essential oil of *Mentha pulegium* L. and *Origanum dictamnus* L.

	**Channel modulation**			**Plant**	
**Terpene**	**TRPA1**	**TRPV1**	**TRPM8**	***Mentha pulegium*** **L**.	***Origanum dictamnus***
Borneol	**X**				
1,8-cineol	**X**		**X**		
Camphor	**X**	**X**	**X**		
Carvacrol	**X**			**X**	**X**
Carvone	**X**	**X**	**X**		
Citral	**X**	**X**	**X**		
Citronellol	**X**	**X**	**X**		
p-cymene	**X**		**X**		**X**
Fenchone	**X**				
Limonene	**X**			**X**	
Linalool	**X**		**X**		**X**
L-menthol	**X**	**X**	**X**		
β-myrcene	**X**	**X**		**X**	**X**
α-pinene	**X**	**X**		**X**	**X**
Pulegone	**X**	**X**	**X**	**X**	
Thymol	**X**		**X**		**X**
Thymoquinone	**X**				

The mark (“**X**”) indicates if the monoterpene listed in the left column modulates a TRP channel activity. Each monoterpene is then associated to its presence (“**X**”) in the essential oil of Mentha pulegium or Origanum dictamnus (right columns).

However, how monoterpenes, particularly based on menthol and pulegone reports, promote analgesic properties through the modulation of the activities of TRPV1, TRPA1, or TRPM8 channels in the setting of inflammatory pain conditions remains to be explored.

## TRP channels in pain, physiologic, and chronic inflammatory conditions

Under normal conditions, nociceptors aim to detect potentially painful stimuli, and the detection and transduction of neuronal messages are less involving: TRPV1, for temperatures > 42°C; TRPM8, for temperatures around 18°C, and TRPA1, for various noxious and mechanical stimuli (Dai, 2016). Aside from these functional arrangements exists an anatomical differentiation: TRPA1-expressing nociceptors are a subset of TRPV1-expressing nociceptors in rodents, whereas TRPM8 remains more expressed in a different set of specific sensory neurons (Dhaka et al., 2008; Jankowski et al., 2017; Kupari et al., 2021). In addition to their sensor roles, it is important to note that TRP channels are also expressed in immune cells [for review (Khalil et al., 2018)]: TRPM8 activity regulates the TNF-α production by macrophage cells (Khalil et al., 2016). These observations allow considering TRP channels not only as peripheral transducers but also as ionic receptors for endogenous ligands and major actors in pain hypersensitivities: TRPM8, TRPA1, and TRPV1 have a fundamental role in the initiation, regulation, and maintenance of hyperalgesia conditions (Schumacher, 2010).

Moreover, they may have a role in pain-central sensitization due to their expression in different regions and/or cellular subtypes of the central nervous system. Indeed, TRPV1 was reported to be expressed in a subset of sensory neurons (Cavanaugh et al., 2011) and central nervous cells (González-Ramírez et al., 2017). Therefore, rodent TRPV1 is present in brain regions such as the sensory, prefrontal, entorhinal, insular, and anterior cingulate cortices, and also the hippocampus, amygdala, and thalamus (for review see Duitama et al., 2020). Interestingly, those regions are involved in pain integration and/or modulation (Ordás et al., 2021; Wahis et al., 2021). However, it is important to note that human TRPV1 was, up to now, only detected in cortical regions (Morelli et al., 2013). The functional contribution of the central expression of TRPV1 is still debated and its contribution to central synaptic plasticity, especially as part of the endocannabinoid system regulation, is under scrutiny (Hakimizadeh et al., 2012; Li et al., 2021). Functional investigations have demonstrated that TRPV1 expressed in hippocampal neurons (Sun et al., 2013) contributes to strengthening spontaneous synaptic transmission (Anstötz et al., 2018).

Functional TRPA1 is described in the hippocampus and somatosensory cortex (Menigoz and Boudes, 2011; Shigetomi et al., 2011; Kheradpezhouh et al., 2017). In the hippocampus, TRPA1 activation increases extracellular GABA concentration, leading to a downregulation of the astrocyte GABA transport GAT-3 activity and a decreased efficiency of inhibitory synapses (Shigetomi et al., 2011). Finally, TRPM8 is present at least in the hypothalamus, septum, and thalamic reticular nucleus (for review Ordás et al., 2021). Interestingly, TRPM8 expressing neurons are innervating the hypothalamus, the periaqueductal gray, and the amygdala regions (Ordás et al., 2021), which are major actors of pain regulation (Eliava et al., 2016; Ordás et al., 2021; Iwasaki et al., 2022).

Under inflammatory conditions, TRP channels are overexpressed at the membrane of sensory nerve endings innervating the inflamed joint. In addition, their intrinsic properties, namely, conductance or sensibility to noxious cues, change and contribute directly to abnormal pain symptoms such as hyperalgesia (Schumacher, 2010). Such conclusions are supported through experimental investigation using inflammatory animal pain models based on the administration of the complete Freund's adjuvant (CFA). Consisting of an injection of CFA into the joint or into the plantar surface of the hind paw, this model is characterized by the presence of exaggerated sensitivity to heat and cold stimulations and heightened sensitivity to mechanical tactile stimulations (Hilfiger et al., 2020, 2021). At the inflammation site, the free endings of the nociceptors are in contact with several inflammatory mediators such as proinflammatory cytokines and protein kinases [for review: (Dai, 2016)]. These interactions lead to sensitization of the nociceptors through different molecular pathways, which contribute directly to pain hypersensitivities and are referred to as “peripheral sensitization” (Mizumura, 1997). Peripheral sensitization allows, for example, the overexpression of TRPV1 in nociceptors (Yu et al., 2008). Such condition was investigated, using complementary pharmacological and genetic approaches. It was demonstrated that TRPV1 is essential to thermal hyperalgesia but not to mechanical hypersensitivity of nociceptors (for review (Dai, 2016). Nonetheless, their activation still recruits mechanical allodynia neuronal circuits (Petitjean et al., 2014). Based on the same approaches, it was clearly shown that TRPM8 is involved in cold hyperalgesia (Colburn et al., 2007; Gong and Jasmin, 2017) while TRPA1 is responsible for mechanical hypersensitivity and nocifensive reactions (Kwan et al., 2006; Eid et al., 2008; Kerstein et al., 2009; Vilceanu and Stucky, 2010; Lennertz et al., 2012; Asgar et al., 2015).

Thus, the three following TRP channels act as a complex molecular system that participates in pain hypersensitivities: TRPV1 contributes to thermal hyperalgesia (Caterina et al., 1997; Petitjean et al., 2014; Koivisto et al., 2022), TRPA1 contributes to thermal, mechanical, and chemical hyperalgesia (Koivisto et al., 2022), and TRPM8 contributes to cold allodynia (Koivisto et al., 2022). Expressed by nociceptors, those channels are easily accessible for exogenous molecules and two paths are generally proposed: a blockage of the channel activity through effective antagonists (Dai, 2016) or, paradoxically, overstimulation leading to long-term desensitization. Therefore, TRPV1, TRPM8, and TRPA1 are ideal targets for analgesic molecules.

## Monoterpenes and TRP channels: An analgesic combination?

Interestingly, both pulegone and menthol act as strong anti-inflammatory molecules in *in vitro* essay with EC_50_ values of 1.2 ± 0.2 and 1.5 ± 0.1 mM, respectively (Hilfiger et al., 2021). In addition, a single intraperitoneal injection of 100 mg/kg pulegone or menthol in CFA rats exerts a transient antihyperalgesic effect on both mechanical, thermal heat and cold hyperalgesia (Hilfiger et al., 2021). Few studies have focused on pulegone-dependent mechanisms that reduce pain reactions. In chicks, intraplantar injection of pulegone causes nocifensive reactions which are attenuated by TRPA1 antagonists (Majikina et al., 2018). In humans and rodents, pulegone appears as a partial agonist of TRPV1 and an inhibitor of the menthol-induced TRPM8 activity (Jabba and Jordt, 2020) (Table 1). These suggest that pulegone is a strong agonist of TRPA1, and a partial TRPV1 agonist, and inhibits the TRPM8 activity. Interestingly, it is proposed that TRPA1 can be desensitized through different cellular pathways which are regulated by the activation of TRPV1 (Akopian et al., 2007; Ruparel et al., 2011; Kistner et al., 2016).

Molecular targets of menthol have been better investigated [for review (Oz et al., 2017)]. In humans and mouse pain models alike, TRPV1 currents activated by heat and capsaicin were inhibited by menthol, a phenomenon that might partially explain its antinociceptive properties (Takaishi et al., 2014); it also activates the TRPM8 to promote an antihyperalgesic effect (Liu et al., 2013). This activation induces a “cooling” sensation, followed by a TRPM8 channel shift into an inactive state that can be reversed only after the wash-out of the agonist compounds: a phenomenon called transient desensitization (Diver et al., 2019; Perri et al., 2020). Furthermore, menthol exerts in murine cells (*in vitro*) a bimodal modulation of TRPA1, activating it at low concentration (0.3–0.7 mM) and blocking it at high concentration (>3 mM) (Lemon et al., 2019). However, in humans, only the activation of TRPA1 by methanol has been observed (Xiao et al., 2008). This suggests that menthol is a strong agonist of TRPM8, and a partial TRPA1 agonist or blocker, and that it inhibits the TRPV1 activity.

In conclusion, both pulegone and menthol strongly interact with TRPM8, TRPA1, and TRPV1 (Table 1) (Takaishi et al., 2014; Oz et al., 2017; Jabba and Jordt, 2020). These pharmacological properties remain to be explored in the setting of TRPV1/TRPA1 channel heterodimers (Fischer et al., 2014). These heterodimers contribute also to pain symptoms (Galindo et al., 2018; Souza Monteiro de Araujo et al., 2020). Based on those properties, monoterpene activating the TRP channel with antihyperalgesic properties may be explained by an initial activation and then desensitization of TRP channels expressed in sensory neurons.

## Prospects of new phytotherapy treatment to alleviate pain conditions

In prospect, beyond plants extracts or essential oils preparations, new treatments relying on specific monoterpenes mixes open an interesting avenue to treat pain. Monoterpenes are very volatile and have the capacity to cross the skin and the blood–brain barrier—thus enabling the modulation of peripheral and central neuronal, and immune cell receptors (Zhang et al., 2015; Weston-Green, 2019).

Based on these properties, commercial topical creams for pain relief were developed, for example, containing a mix of 3 monoterpenes: camphor, 1,8-cineole, and menthol (product NPN 02248563 RUB-A533 Antiphlogistine). To define the efficiency of a new putative mix, for new topical preparations, for instance, the reassessment of compounds found in traditional/popular medicine treatments and investigation of other bioactive principles that could alleviate chronic pain conditions seems a promising path. With an aim to develop a local and effective treatment to alleviate chronic conditions, this work intends to demonstrate the potential for the discovery of new antinociceptive drugs: there are already 50,000 terpenes identified (Breitmaier, 2006; Yamada et al., 2015) and their putative synergistic analgesic effects remain to be explored. This approach will further enable us to discover new uses for local plant species and to consider meaningful actions toward their valorization on environmental and patrimonial levels. Therefore, efforts toward the translation, annotation, and promotion of old folk books to a large audience are still worthy enterprises [for review (UNESCO World Heritage Center, 2010)]. Although ethnobotanical studies have been booming since the 1980s, few initiatives managed to share the results with traditional communities or raise awareness of local plant materials (Reyes-García et al., 2021; Schultz et al., 2021).

In the era of open science and open data sharing and within the logic of the “one health” theory, it is of the utmost importance to share this knowledge with citizens and professionals and to protect our plant heritage increasing our global commitment to biodiversity, as advised by international organizations (UNESCO, 2018; Mackenzie and Jeggo, 2019). We believe that this approach will bring new virtuous and resilient cycles of innovation, while increasing citizens' commitment and engagement toward their ecosystems.

## Author contributions

EH performed the plant databases analysis. HP and LH performed the monoterpene databases analysis. EH, HP, and AC coordinated and produced the review. All authors discussed and elaborated the ideas, wrote the first draft, corrected it until the final version was obtained, and approved the manuscript.

## Funding

This work was supported by the Centre National de la Recherche Scientifique contract UPR3212, the Université de Strasbourg contract UPR3212, the Graduate School of Pain EURIDOL, ANR-17-EURE-0022, and the ANRT CIFRE Grants No. 2018/1140 (to AC and HP) and no. 2016/047 (to HP).

## Conflict of interest

HP, EH, LH, and GR are salaried employees at the private company Benephyt. The remaining authors declare that the research was conducted in the absence of any commercial or financial relationships that could be construed as a potential conflict of interest.

## Publisher's note

All claims expressed in this article are solely those of the authors and do not necessarily represent those of their affiliated organizations, or those of the publisher, the editors and the reviewers. Any product that may be evaluated in this article, or claim that may be made by its manufacturer, is not guaranteed or endorsed by the publisher.
